# Association of elevated glycated hemoglobin and obesity with afternoon napping for more than 1 h in young and middle-aged healthy adults

**DOI:** 10.3389/fpsyt.2022.869464

**Published:** 2022-10-10

**Authors:** Mohammed A. Al-Abri, Ibtisam Al Lawati, Fahad Al Zadjali

**Affiliations:** ^1^Department of Physiology, College of Medicine and Health Sciences, Sultan Qaboos University, Muscat, Oman; ^2^Department of Physiology, Oman College of Health Sciences, Muscat, Oman; ^3^Department of Biochemistry, College of Medicine and Health Sciences, Sultan Qaboos University, Muscat, Oman

**Keywords:** napping, sleep, diabetes mellitus, glycated hemoglobin, obesity

## Abstract

**Introduction:**

Sleep has different patterns followed worldwide and can be influenced by social, cultural, and environmental factors. Daytime napping is commonly practiced in different parts of the world with controversial results of its effect on glucose metabolism. The current study aims to examine the association of afternoon napping and night sleep duration with metabolic derangements.

**Methods:**

This is a cross-sectional study involving young adults and middle-aged subjects. Anthropometric measurements were taken for height and weight and hip and waist ratio. Consented subjects were asked to wear actigraphy for 1 week and run their usual daily activities. Home sleep apnea testing was performed to exclude obstructive sleep apnea. Subjects had been asked to come fasting on day seven for blood collection to test for fasting glucose, glycated hemoglobin, lipid profile, and insulin.

**Results:**

A total of 405 subjects were involved to complete the study (52% male, 48% female). The mean age of participants was 32.8 ± 11.5 years. The study indicated that the duration of afternoon napping was significantly associated with abnormal glycated hemoglobin (HbA1c > 5.7%) (*p* = 0.01) and body mass index (*p* = 0.046) independent of age, gender, and nocturnal sleep duration. Nocturnal sleep duration was associated with increased insulin level (*p* = 0.04).

**Conclusion:**

Afternoon napping is associated with an increased level of glycated hemoglobin and obesity and that may predispose to the development of type 2 diabetes mellitus.

## Introduction

Sleep is an imperative physiological aspect that maintains the circadian rhythm and hence a healthy life. Sleep patterns are identified according to individual satisfaction, specific sleep timing, sufficient duration, high efficiency, and continuous alertness throughout one's waking hours. Reduced sleep duration and quality are becoming endemic in many societies because of lifestyle changes and modernization and chronic insufficient sleep is considered a public health problem (1). Obesity and cardiometabolic risk are on the rise worldwide (2, 3) and the underlying etiology of this pandemic is multifactorial, with well-recognized contributions from the diet (large portion sizes, nutrient-dense foods, and others) and physical inactivity (3, 4). However, such factors alone do not explain the significant epidemic of obesity and cardiometabolic diseases and sleep duration has a strong confounding factor.

The link between sleep and metabolism is well established for a long time and that includes mainly the association between sleep duration and timing and cardiometabolic diseases (5, 6). Studies indicated the association between chronic sleep restriction and poor sleep quality and the increased risk of obesity, insulin resistance, hypertension, and lipid abnormalities in different age populations. Nevertheless, several epidemiologic studies have reported that diabetes risk is elevated in both short (6, 7) and long (8) sleepers after adjusting for confounding risk factors, including waist girth. Therefore, it is a U-shaped relationship that shows an association between sleep duration and cardiometabolic risk, independent of sleep pattern. Afternoon napping also known as “siesta” is a common practice in some parts of the world, particularly in hot areas, such as the middle east (8). Relationships between napping and other health outcomes, such as diabetes, have been even less well characterized by debatable results about the duration of the napping and the metabolic effect (9–12). A study in Mediterranean islands indicated that daytime napping is associated with increasing metabolic syndrome (MetS) and frequent “siesta” was positively linked to the odds of MetS presence in women but not in men (13). Short daytime napping duration (<1 h) is associated with a reduced rate of metabolism-related diseases and it may have a protective effect on people from negative health conditions, whereas long daytime napping duration is associated with a higher prevalence of diabetes, and therefore may predispose to unfavorable health outcomes (14).

We have earlier reported that people may adapt to different sleep patterns, which could be multiple episodes per 24 h with variable durations (15). Moreover, we found that the majority of participants practiced afternoon napping (85%) with a mean duration of 45 ± 43 min (15). Earlier reports found that the prevalence of napping reported in surveys and in diaries ranges from ~10–65% (16, 17).

Despite conflicting literature on the associations between sleep duration and timing with obesity and metabolic risk, normal sleep is being explored as a possible factor to minimize the risk of cardiovascular and metabolic morbidities. To address these issues, we measured sleep duration using actigraphy, anthropometry, and markers of metabolic syndrome. Specifically, we aimed to determine the link between long afternoon napping (>1 h) and metabolic abnormalities. We also aimed to determine if associations existed between night sleep duration and traits of metabolic components: lipid profile, blood pressure, hyperglycemia, insulin resistance, and adiposity. In the present study, we hypothesized that long afternoon napping of more than 1 h and in presence of a normal night, sleep duration is associated with an increased risk of metabolic diseases.

## Methods

### Study design and sample size

This was a cross-sectional descriptive community-based study. The Medical Research Ethics Committee at Sultan Qaboos University approved the study design and methods with code MREC # 1078 and the study was conducted in accordance with the declaration of Helsinki. All participants were asked to sign a written consent before enrolling in the study.

The sample size was determined based on previous literature reviews that study the cardiometabolic risks in siesta vs. nonsiesta sleep patterns. The mean, standard deviation, and mean difference of five quantitative parameters were collected including, glycated hemoglobin, fasting blood glucose (FG), total cholesterol (TC), triglycerides (TG), and BMI (BMI). The target group was healthy local adults' men and women, aged 18–64 years. An adequate sample size of 400 subjects was estimated based on the prevalence of event of interest (50%), with a margin of error of 10% on either side of the estimate with 95% confidence intervals with a prior estimate that 90% of participants would have the siesta habit, the calculated sample size would achieve a power of 80%.

Participants were recruited through a variety of methods, including press releases, emails, brochure distribution to local schools, government establishments, and community events. The study was conducted in Muscat, the capital city and the largest metropolitan area in Oman from January 2016 to February 2018. Subjects were excluded if they met any of the following criteria: (1) past/present medical history including cancer, any psychiatric illness affecting his sleep behavior including anxiety and depression, (2) shift workers, (3) subjects with moderate and severe obstructive sleep apnea (OSA) are defined by apnea-hypopnea index (AHI) > 15 (18, 19), (4) Pregnant and breast-feeding women were also excluded as well as mothers who have children below 1 year of age, (5) The participants who had not traveled across the time zone for at least 1 month before enrolling in study, (6) Smokers and regular alcohol drinkers, and (7) people with an established diagnosis of type 2 diabetes mellitus (T2DM) and/or hypertension. The Muslim's holy month of Ramadan and the following month in the lunar calendar were excluded because of changes in people's rituals, sleep, and eating habits.

### Measurement of sleep patterns

An actigraphy watch (SOMNOWATCH TM Plus, SOMNO medics, Germany, 2014) was used in this study. The participants were requested to wear the actigraphy watch on the dominant arm for 7 days 24 h a day except during showering. They were also given an activity diary at the same time for confirmation. The diary includes working, eating and resting time, and physical activities. The participants were asked to run their daily activities as usual and avoid traveling across time zone during the period of the study. The variation in night sleep duration and afternoon napping was identified and the average of 1 week was considered for the statistical analysis. Subjects were also provided with home sleep apnea testing (HSAT) (AASM level III) (Somnotouch, Somnomedics, Germany, 2015) for one night to exclude obstructive sleep apnea (20). Actigraphy recordings were downloaded and analyzed by the researcher and the timing of sleep and wake up and afternoon napping was obtained (21). Nocturnal sleep duration and afternoon napping were also calculated. Afternoon siesta was categorized into two groups: short siesta (<60 min) and long siesta (22).

All potential confounders were also identified a priori and that include age, gender, diet, level of exercise, level of education, and socioeconomic status, all of which are known to be risk factors for cardiometabolic diseases and it is already reported in previously published articles (15, 23). All participants reported a carbohydrate-rich diet and no or mild (one episode of walking/ week) exercise.

### Anthropometric measurements and fasting blood collection

The participants were informed to come on day 8 early morning fasting for blood collection. After ensuring 12 h of overnight fasting, 15 ml of blood (butterfly blue needle of 23 gauge, BD, USA) was collected from each participant between 6 and 10 am. The biochemical investigations were measured in a clinical laboratory (The analysis is performed on the Roche COBAS INTEGRA 400 analyzer, Germany) and that includes fasting glucose (FG), HbA1c, serum insulin level, total cholesterol (TC), high-density lipoprotein (HDL), low-density lipoprotein (LDL), and triglycerides (TG).

Before blood collection, anthropometric measurements were taken, which included weight (kg), height (cm), hip and waist circumferences during the first visit (day 1) and the second visit (day 8). The weight was measured by a digital weighing scale (Seca, Equip Medical, Singapore). Blood pressure was measured in a sitting position using an appropriate-sized cuff with a blood pressure unit (SureSigns VM8, Philips, Germany), and it was measured two times in two different visits and the average of the two readings was taken for the statistical analysis. All participants were asked to fill in the Arabic version of the Epworth sleepiness questionnaire (ESS) to assess for daytime sleepiness (24).

### Outcome ascertainment

There was a total of six metabolic outcomes in the present study and that include, obesity, elevated fasting glucose, HbA1C, HDL, LDL, and TG. Metabolic syndrome was defined according to the Joint Interim Statement of the International Diabetes Federation (IDF) (25). Obesity was defined as hip/waist and waist circumference over 80 cm in women and over 90 cm in men and body mass index (BMI) was classified according to the world health organization (WHO) classifications (26).

Participants were classified as normal HbA1c <5.7%, pre-diabetes 5.8–6.4% and type 2 diabetes > 6.5%. (FPG < 5.6 mmol/L), IFG (5.6 ≤ FPG < 7.0 mmol/L), and diabetes (FPG ≥ 7.0 mmol/L) based on local laboratory guidelines. Lipid profile that includes HDL, LDL, and TG were classified according to the latest guidelines of lipid treatment guidelines in Oman (27).

### Statistical analysis

Descriptive analysis for continuous variables was used to calculate mean, median, and standard deviation (SD). Prevalence and frequencies were expressed as percentages. The *t*-test was used to find the mean difference between the two groups (short and long siesta). Regression analysis was performed to correlate sleep parameters (night sleep durations in hours and siesta duration in minutes) and metabolic components. A *p*-value (two-tailed) of <0.05 was considered statistically significant. All data were coded and analyzed with the Statistical Package for Social Sciences Software (SPSS Version 22.0, Inc., Chicago, IL, USA). Participants who fully completed the study were included in the data analysis. Non-responders or those with incomplete actigraphy data (he/she used the watch for <1 week) were excluded. Additionally, subjects with AHI>15 are also excluded.

## Results

### Demographics of the study population

Around 800 people were approached and only 405 subjects completed the study (52% male, 48% female) with a response rate of 50.6%. The mean age of the subjects was 32.82 ± SD11.5years and BMI 26.0 ± 7.0 kg/m^2^. The mean night sleep duration is 6.9 ± SD 1.33 h and the mean afternoon napping was 45.56 ± SD 43.18 min. The most frequent bed time of the study sample was between 11:00 pm and 12:00 am and the frequent final wake up time was between 6 and 7:00 am. More demographic data are listed in Table 1.

**Table 1:** Demographics of the study sample (*n* = 405).

	**Mean**	**SD**
Age (years)	32.7	11.5
BMI (Kg/m^2^)	26.0	7.0
Exercise duration (min/week)	130.5	200.8
Epworth sleepiness scale	8.2	4.0
Siesta duration (min)	45.6	43.2
Night sleep duration (hours)	6.9	1.3
Total 24-day sleep (hours)	7.9	1.5

BMI, body mass index.

### Afternoon napping and metabolic components

Table 2 shows a comparison between short and long afternoon napping with different metabolic syndrome traits. Results showed significant difference in HbA1c between short nap (mean = 5.38 ± 0.71%) and long afternoon nap (mean = 5.63 ± 1.19%) (*p* = 0.02) and in BMI; short nap (mean = 25.47 ± 7.08 kg/m^2^) and long afternoon nap (mean = 29.90 ± 6.87 kg/m^2^) (*p* = 0.049). Other biochemical parameters did not show any significant association with an afternoon nap. We further adjusted the statistics for the following confounding variables: age, exercise durations, night sleep duration, and total 24-h sleep duration. Afternoon nap duration was significantly correlated with HbA1c (*p* = 0.01) (Table 3), and BMI (*p* = 0.046) (Table 4) after adjusting to night sleep duration, age and gender. Other glycemic parameters did not show any significant association with an afternoon nap (*p* > 0.05) (Table 5).

**Table 2:** Metabolic comparison between short and long siesta (*n* = 405).

	**Short siesta**	**Long siesta**	***P*-value**
Insulin (mIU/L)	8.5 ± (6.8)	9.0 ± (8.1)	0.53
HbA1c (%)	5.4 ± (0.7)	5.6 ± (1.2)	0.02
BMI (kg/m^2^)	25.5 ± (7.1)	26.9 ± (6.9)	0.049
Waist circumference (cm)	86.6 ± (16.7)	88.3 ± (15.6)	0.29
Fasting glucose (mmol/L)	5.5 ± (1.4)	5.5 ± (1.5)	0.7
Total cholesterol (mmol/L)	4.8 ± (1.0)	4.8 ± (0.9)	0.92
Triglycerides (mmol/L)	1.2 ± (0.7)	1.1 ± (0.6)	0.64
HDL (mmol/L)	1.3 ± (0.4)	1.3 ± (0.4)	0.24
LDL (mmol/L)	2.9 ± (0.9)	3.0 ± (0.9)	0.53

Short Siesta <60 min of siesta sleep. Long Siesta: >60 min of siesta sleep. *P*-value, Student t test. HbA1c, Glycated hemoglobin BMI: Body mass index; HDL, high density lipoprotein; LDL, low density lipoprotein.

**Table 3:** Linear regression analysis of HbA1C (%) with different confounding variables.

**Independent variables**		**Standardized coefficients**	***P* value**	**95.0% confidence interval for B**	
		**beta**		**Lower bound**	**Upper bound**
1	(Constant)		0.00	3.90	5.01
	Gender	−0.04	0.46	−0.24	0.11
	Age (years)	0.39	0.00	0.02	0.04
	Night sleep duration (h)	0.005	0.91	−0.06	0.07
	Siesta (min)	0.14	0.003	0.001	0.005

Dependent variable: HbA1C%.

**Table 4:** Linear regression analysis of BMI (kg/m^2^) with different confounding variables.

**Independent variables**		**Standardized coefficients**	***p* value**	**95.0% confidence interval for B**	
		**beta**		**Lower bound**	**Upper bound**
1	(Constant)		0.000	17.31	25.80
	Night sleep duration (h)	0.025	0.579	−0.34	0.61
	Siesta (min)	0.091	0.044	0.00	0.03
	Gender	−0.194	0.000	−4.03	−1.42
	Age (years)	0.342	0.000	0.15	0.26

Dependent variable: BMI (Kg/m^2^).

**Table 5:** Regression analysis of afternoon napping (min) with metabolic components after adjustment for age, exercise and night sleep duration.

	***P*-value**	**Standardized coefficients**	**95% confidence interval for beta coefficient**	
		**beta**	**Lower bound**	**Upper bound**
Triglycerides (mmol/l)	0.810	0.01	−0.001	0.001
LDL (mmol/l)	0.441	0.04	−0.001	0.003
HDL (mmol/l)	0.157	−0.07	−0.002	0.000
Fasting glucose (mmol/l)	0.852	−0.009	−0.003	0.002
Insulin (mIU/L)	0.760	−0.02	−0.018	0.013
HbA1C%	0.01	0.12	0.001	0.004
BMI (kg/m^2^)	0.046	0.09	0.000	0.029
Waist circumferences (cm)	0.324	0.04	−0.015	0.046
Hip/waist	−0.058H	0.09	0.000	0.000
HOMA-IR	0.16	0.08	0.000	0.001

HbA1c, Glycated hemoglobin BMI, Body mass index; HDL, high density lipoprotein; LDL, low density lipoprotein; HOMA-IR, Homeostatic Model Assessment of Insulin Resistance.

### Night sleep duration and metabolic components

We then assessed the association of night sleep duration with metabolic traits. Regression analysis (Table 6) indicates that night sleep duration was associated with increased insulin level (*p* = 0.04) and hip/wait (*p* = 0.03). Lipid profile parameters of TG (*p* = 0.62) and LDL (*p* = 0.50) did not show any correlation except HDL (*p* = 0.04). Furthermore, HbA1c, fasting glucose, and BMI did not show any significant association with night sleep duration (*p* > 0.05) after adjusting for an afternoon siesta, age, and total 24-h day sleep.

**Table 6:** Regression analysis of night sleep duration with metabolic components after adjustment for age and afternoon siesta (napping).

	***P*-value**	**Standardized coefficients**	**95% confidence interval for beta coefficient**	
		**beta**	**Lower bound**	**Upper bound**
Triglycerides (mmol/l)	0.62	0.03	−0.03	0.06
LDL (mmol/l)	0.50	0.03	−0.05	0.09
HDL (mmol/l)	0.04	0.10	0.002	0.06
Fasting glucose (mmol/l)	0.99	−0.02	−0.09	0.09
Insulin (mIU/L)	0.04	0.11	0.04	1.03
HbA1C%	0.75	0.02	−0.05	0.07
BMI (kg/m^2^)	0.91	0.01	−0.43	0.49
Waist circumferences (cm)	0.71	−0.02	−1.19	0.82
Hip/waist	0.03	−0.10	−0.01	−0.001
HOMA-IR	0.32	0.06	−0.008	0.02

HbA1C, Glycated hemoglobin BMI, Body mass index; HDL, high density lipoprotein; LDL, low density lipoprotein; HOMA-IR, Homeostatic Model Assessment of Insulin Resistance.

### Total 24-h sleep and metabolic components

Table 7 shows that total 24-h sleep was significantly associated with TG (*p* = 0.04) but not with other biochemical parameters (*p* > 0.5) nor with obesity parameters (BMI, h/w, and waist circumference, *p* > 0.05).

**Table 7:** Regression analysis of total 24-h sleep duration with metabolic components after adjustment for age and afternoon siesta (napping) and night sleep duration.

		***P* value**	**Standardized coefficients**	**95% confidence interval for B**	
			**beta**	**Lower bound**	**Upper bound**
Triglycerides (mmol/l)		0.04	0.27	0.01	0.24
LDL (mmol/l)		0.36	−0.10	−0.2	0.07
HDL (mmol/l)		0.77	−0.03	−0.06	0.05
Fasting glucose (mmol/l)		0.56	0.06	−0.15	0.27
Insulin (mIU/L)		0.92	0.01	−1.02	1.13
HbA1C%		0.29	−0.13	−0.23	0.07
BMI (kg/m^2^)		0.12	−0.16	−1.73	0.19
Waist circumferences (cm)		0.58	−0.05	−2.57	1.43
Hip/waist		0.25	−0.12	−0.02	0.01
HOMA-IR		0.29	1.03	−0.12	0.41

HbA1C, Glycated hemoglobin; BMI, Body mass index; HDL, high density lipoprotein; LDL, low density lipoprotein; HOMA-IR, Homeostatic Model Assessment of Insulin Resistance.

## Discussion

This cross-sectional descriptive study was conducted among the Arab population in Oman using an objective method of actigraphy to measure sleep duration and timing. We have indicated earlier that people in Oman and other similar communities have different sleep patterns which involve habitually long afternoon napping that may extend to more than 1 h (15, 23, 28). The current study indicated that long afternoon napping of more than 60 min is associated with increased glycated hemoglobin (HbA1C) and obesity in subjects with normal nocturnal sleep duration.

Our findings support the hypothesis that a long afternoon siesta is associated with abnormal glycemic control and adapting certain sleep patterns that involved long afternoon napping may predispose to metabolic abnormalities. The evidence for daytime napping and metabolic abnormalities from previous studies was inconclusive with variable results about the duration and frequency of the nap. Although this study is cross-sectional, it objectively measured the sleep duration using actigraphy, unlike previous studies that used questionnaire-based surveys which are subjected to re-call bias (29). Previous studies concerning the health impact of napping examined the relationship with T2DM. One study found that afternoon nap duration was associated with the risk for T2DM, and longer nap duration (60–90 min) was related to an increased risk for impaired fasting plasma glucose (11). Nevertheless, they used self-report sleep habits and the average age of their cohort was 50 years. In our study, we used a more objective method and we ruled out moderate and severe OSA as a confounder to draw our conclusion. Moreover, we studied a relatively younger population with an average age of 32 years. Another study by the Guangzhou group found that napping for more than 30 min in a population older than 50 years is associated with T2DM (30). A prospective study reported that daytime napping of more than 30 min and after an average of 4.5 years of follow-up, was significantly associated with an increased risk of elevated HbA1c level (>6.5%) in men and women in the Chinese population (31). Our sample did not reveal nocturnal sleep deprivation with an average night sleep duration at almost 7 h (32) but almost all participants practiced afternoon siesta with variable duration. Therefore, our findings are in agreement with the prospective study and in absence of nocturnal sleep deprivation.

Our findings could be explained by the fact that long afternoon napping of more than an hour may alter the circadian rhythm and that would indirectly alter the link between healthy sleep and normal metabolism of carbohydrates (33). Long afternoon napping would shift night bed and rise timing toward late phase sleep and this may cause circadian misalignment (34).

It is important to mention that night sleep duration is correlated with insulin level and obesity by hip/waist and that may add to the burden of metabolic derangement if the subject goes for afternoon napping. Nevertheless, the total 24-h day of sleep did not correlate significantly with any metabolic parameters except for TGA and that might be attributed that the majority of young people in Oman tend to have late dinner. We could assume at this point that afternoon napping with normal night sleep duration is independently associated with glycemia abnormalities.

A major limitation of this study is the nature of the study itself as with cross-sectional analysis you cannot infer causality; indeed, afternoon napping may be a marker of unfavorable health outcomes and lifestyle traits, which is accounted for to some extent by adjusting for measured confounders. Nevertheless, doing a prospective long-term study with objective methods of actigraphy and home sleep study would be cumbersome and incur a bigger cost. The strength of our study is the objective method of measuring sleep and all participants were screened for OSA using HSAT. Another limitation of the study is the possible impact of diet which is not fully addressed in this study, particularly the quantity of carbohydrate consumption which is a major dietary component among the local population. Furthermore, the confounding factor of exercise should also be fully investigated in terms of duration and frequency and that would add more strength to any study associating afternoon siesta and metabolism.

## Conclusion

Afternoon napping of more than 1 h is correlated with increased glycated hemoglobin and obesity in participants with normal night sleep duration in young and middle-aged adults. Night sleep duration is correlating with insulin level but total 24-h day sleep did not correlate with any glycemia parameters or obesity. Therefore, long napping of more than 1 h could be considered a risk for developing T2DM. It would be recommended for public health workers and physicians to educate people to avoid long napping duration, especially in societies with a high prevalence of obesity and diabetes (3, 35).

## Data availability statement

The raw data supporting the conclusions of this article will be made available by the authors, without undue reservation.

## Ethics statement

The studies involving human participants were reviewed and approved by Ethics Committee, College of Medicine and Health Sciences, Sultan Qaboos University, Muscat, Oman. The patients/participants provided their written informed consent to participate in this study.

## Author contributions

All authors listed have made a substantial, direct, and intellectual contribution to the work and approved it for publication.

## Funding

This study was funded by the research council of Oman Project No. RC/MED/PHYS/15/01.

## Conflict of interest

The authors declare that the research was conducted in the absence of any commercial or financial relationships that could be construed as a potential conflict of interest.

## Publisher's note

All claims expressed in this article are solely those of the authors and do not necessarily represent those of their affiliated organizations, or those of the publisher, the editors and the reviewers. Any product that may be evaluated in this article, or claim that may be made by its manufacturer, is not guaranteed or endorsed by the publisher.
